# Improvement of surgical complications using single-lumen endotracheal tube intubation and artificial carbon dioxide pneumothorax in esophagectomy: a meta-analysis

**DOI:** 10.1186/s13019-021-01459-1

**Published:** 2021-04-21

**Authors:** Kai-Hao Chuang, Hsing-Hua Lai, Yu Chen, Li-Chun Chen, Hung-I Lu, Yen-Hao Chen, Shau-Hsuan Li, Chien-Ming Lo

**Affiliations:** 1 Department of Thoracic & Cardiovascular Surgery, Kaohsiung Chang Gung Memorial Hospital and Chang Gung University College of Medicine, 123 Ta-Pei Road, Niaosung Dist., Kaohsiung, Taiwan, Republic of China; 2 Department of Hematology-Oncology, Kaohsiung Chang Gung Memorial Hospital and Chang Gung University College of Medicine, Kaohsiung, Taiwan

**Keywords:** Artificial pneumothorax, CO_2_, Esophagectomy, Video-assisted thoracoscopic surgery

## Abstract

**Background:**

Esophageal cancer has a poor prognosis. Surgery is the main treatment but involves a high risk of complications. Some surgical strategies have tried to eliminate complications. Our meta-analysis tried to find the benefits of single-lumen endotracheal tube intubation with carbon dioxide (CO_2_) inflation.

**Methods:**

A systematic search of studies on esophagectomy and CO_2_ inflation was conducted using PubMed, Medline, and Scopus. The odds ratio of post-operative pulmonary complications and anastomosis leakage were the primary outcomes. The standardized mean difference (SMD) in post-operative hospitalization duration was the secondary outcome.

**Results:**

The meta-analysis included four case-control studies with a total of 1503 patients. The analysis showed a lower odds ratio of pulmonary complications in the single-lumen endotracheal tube intubation in the CO_2_ inflation group (odds ratio: 0.756 [95% confidence interval, CI: 0.518 to 1.103]) compared to that in the double-lumen endotracheal tube intubation group, but anastomosis leakage did not improve (odds ratio: 1.056 [95% CI: 0.769 to 1.45])). The SMD in hospitalization duration did not show significant improvement. (SMD: -0.141[95% CI: − 0.248 to − 0.034]).

**Conclusions:**

Single-lumen endotracheal tube intubation with CO_2_ inflation improved pulmonary complications and shortened the hospitalization duration. However, no benefit in anastomosis leakage was observed.

## Background

Esophageal cancer has poor overall survival because of delayed diagnosis. Surgery (esophagectomy) followed by chemoradiotherapy is the main treatment strategy. However, the surgical procedure will carry a high risk of morbidity such as pulmonary complications, anastomosis leakage, and long hospitalization duration.

Surgeons are interested in decreasing post-operative complications and have developed methods to improve outcomes. Some studies focused on the anesthesia method with single-lumen endotracheal tube intubation with artificial pneumothorax induced by carbon dioxide (CO_2_) inflation. The first published single lumen intubation compared double lumen intubation anethesia in esophagectomy is Ruixiang Zhang et. al. in Interactive Cardiovascular and Thoracic Surgery 2014 [1]. We knew single lumen intubation is benefit in minimal invasive esophagectomy to approach lymph nodes around left recurrent laryngeal nerve and some published studies adopted this surgical intervention [2]. However, some hospital did not familiar with this method. Some literature even suggested carbon dioxide insufflation is not necessary such as Fernando A Herbella et. al in Word Journal of Gastroenterology 2010 [3]. Although double lumen intubated for anethesia with one lung ventilation could get better surgical field, hoarsness, tracheal injury and lung injury induced by one lung ventilation may get worse result after operation. In current clinical practice, minimally invasive esophagectomy performed by single lumen intubation is a trend to get better exposure of left recurrent laryngeal nerve injury. However, it remains some debate between using carbon dioxide insufflation or not. Our meta-analysis tried to compare single-lumen endotracheal tube intubation anesthesia plus CO_2_ inflation with double-lumen endotracheal tube intubation.

We present the following article in accordance with the PRISMA reporting checklist.

## Methods

### Search strategy and inclusion criteria

PubMed, Medline, and Scopus were searched for studies with keywords including “artificial pneumothorax or CO_2_” and “esophagectomy or VATS.” A total 136 results were found. We excluded literature which were not written in English and were not human studies. We also excluded robotic surgery, case reports, and literature reviews. Studies on minimally invasive esophagectomy using single-lumen intubation with CO_2_ inflation were included.

All included studies were case control studies. All retrieved studies were required to include two treatment arms. One was CO_2_ inflation for induction of artificial pneumothorax and intubated with single-lumen endotracheal tube. The other was traditional one-lung ventilation by double-lumen endotracheal tube intubation. The target population included patients diagnosed with esophageal cancer.

### Data extraction and quality assessment

Three reviewers critically read all the studies that were included in our analysis and extracted the data. We recorded the year, first author, number of treatment arms, and results concerning three different parameters, including pulmonary complications, anastomosis leakage, and hospitalization duration. The quality of enrolled studies was evaluated by the two reviewers using the Newcastle-Ottawa Scale. The scale includes three parts for the case control study, namely, “SELECTION” (4 items), “COMPARABILITY” (1 item), and “EXPOSURE” (3 items). Disagreements between reviewers were discussed by other authors and the corresponding author.

### Data synthesis and analysis

The odds ratios (ORs) of post-operative pulmonary complications and anastomosis leakage in the single-lumen endotracheal tube intubation in the CO_2_ artificial pneumothorax group (SLET group) compared with those in the double-lumen endotracheal tube intubation group (DLET group) were the primary outcomes. The standardized mean difference (SMD) in post-operative hospitalization duration in SLET group compared with that in the control group comprised the secondary outcome. A random effects model was employed to pool individual SMDs and ORs. The heterogeneity was determined by I_2_ tests, in which values > 50% were regarded as obvious heterogeneity. Potential publication bias was examined by Egger’s test and Funnel plots. Statistical significance was defined as *p*-value < 0.05. All statistical analyses were performed using Comprehensive MetaAnalysis software, version 3 (Biostat, Englewood, NJ, USA).

### Ethical statement

The authors are accountable for all aspects of the work in ensuring that questions related to the accuracy or integrity of any part of the work are appropriately investigated and resolved.

This article used published accessible literature without containing deeply personal, sensitive, or confidential information from participants. Therefore, institutional review board approval is not necessary.

## Results

### Study search and characteristics of included patients

**Fig. 1:**
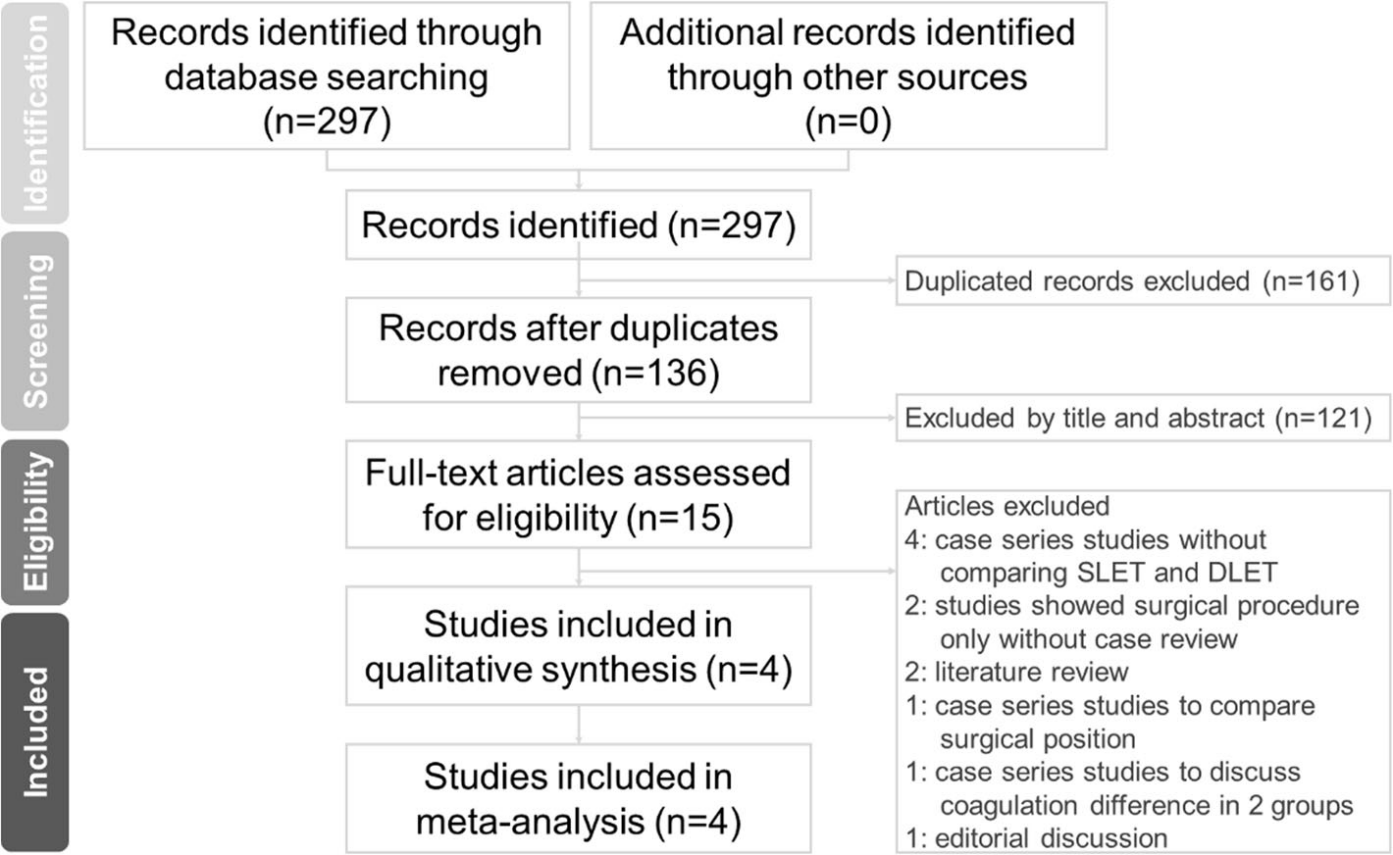
Preferred reporting items for systematic reviews and meta-analyses (PRISMA) flow diagram for the searching and identification of included studies

We searched the databases and retrieved 136 results. After reviewing their title, abstract, and keywords, 15 papers were selected for meticulous commentary by the reviewers. We excluded papers which did not meet our inclusion criteria (Fig. 1). Four studies were excluded because they were case series that did not compare CO_2_ inflation and traditional one-lung ventilation [4–7]. Two studies were excluded because one introduced a surgical technique with CO_2_ inflation in esophagectomy [8], and the other showed a video-assisted thoracoscopic surgical procedure [9]. One study was excluded because it was a case control study that compared different surgical positions [10]. One study was an editorial discussion [11]. One study discussed about differences in coagulation between DLET and SLET groups [12]. Two studies were literature reviews [13, 14]. Finally, four case control studies were included in this meta-analysis [1, 15–17].

The final quantitative analysis included 915 patients in the DLET group and 588 patients in the SLET group. The age ranges were from 53.91 to 76.9 years in the DLET group and 53.48 to 80 years in the SLET group. Patient characteristics, study methodology, and quality assessment of included trials are listed in Table 1. The detail of quality assessment of included trials is listed in Table 2.

**Table 1:** Patient characteristics, study methodology, and quality assessment of included trials

Author, year	Patient Diagnosis	Surgery	Study Design	Enrolled sample number (Male/Female)	Average age, years	Intention-to-treat	Outcome measurement	Quality assessment
Ruixiang Zhang et.al, 2014 [1]	Esophageal cancer	MIE	Case-Control	DLET:60/21 SLET:34/8	DLET:64.3 ± 6.5 SLET:62.5 ± 6.5	Not mentioned	Surgical variables and postoperative complications	8
Itasu Ninomiya et.al, 2017 [15]	Esophageal cancer	MIE	Case-Control	DLET:49/9 SLET:28/9	DLET: 65.8 ± 8.5 SLET:63.1 ± 8.2	Not mentioned	Postoperative mortality and morbidity rates; Surgical outcomes during thoracic procedures	8
Miao Lin et.al, 2018 [16]	Esophageal cancer	MIE	Case-Control	DLET:527/178 SLET:359/102	DLET:61.8 ± 7.89 SLET:61.4 ± 7.92	Not mentioned	Surgical variables and postoperative complications; Parameters during surgery	8
Shinsuke Nomura et.al, 2020 [17]	Esophageal cancer	MIE	Case-Control	DLET:59/12 SLET:41/7	DLET:71.1 ± 5.8 SLET:69.9 ± 10.1	Not mentioned	Comparison of postoperative outcomes; Perioperative changes in SIRS criteria, PaO_2_/FiO_2_ ratio and CRP	8

*MIE* Minimally invasive esophagectomy, *DLET* Double lumen endotracheal tube intubation, *SLET* Single lumen endotracheal tube intubation, *SIRS* Systemic inflammatory response syndrome

**Table 2:** Details of quality assessment of the included trials

Author, year	Selection				Comparability Comparability of cases and controls on the basis of the design or analysis	Exposure			Quality assess-ment
	Is the case definition adequate?	Re-presentativeness of the cases	Selection of Controls	Definition of Controls					
						Ascertainment of exposure	Same method of ascertainment for cases and controls	Non-Response rate	
Ruixiang Zhang et.al, 2014 [1]	*	*		*	**	**	*		8
Itasu Ninomiya et.al, 2017 [15]	*	*		*	**	**	*		8
Miao Lin et.al, 2018 [16]	*	*		*	**	**	*		8
Shinsuke Nomura et.al, 2020 [17]	*	*		*	**	**	*		8

### Pooled odds ratio of pulmonary complication, anastomosis leakage, and SMDs in post-operative hospitalization duration

**Fig. 2:**
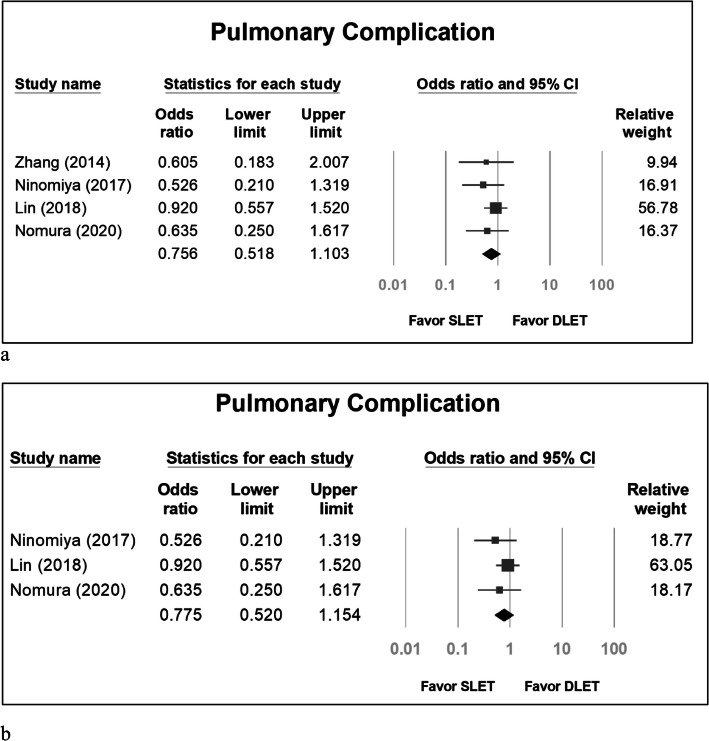
Pulmonary complications between the DLET group and SLET group, included all studies (**a**) and excluding Zhang’s series (**b**)

**Fig. 3:**
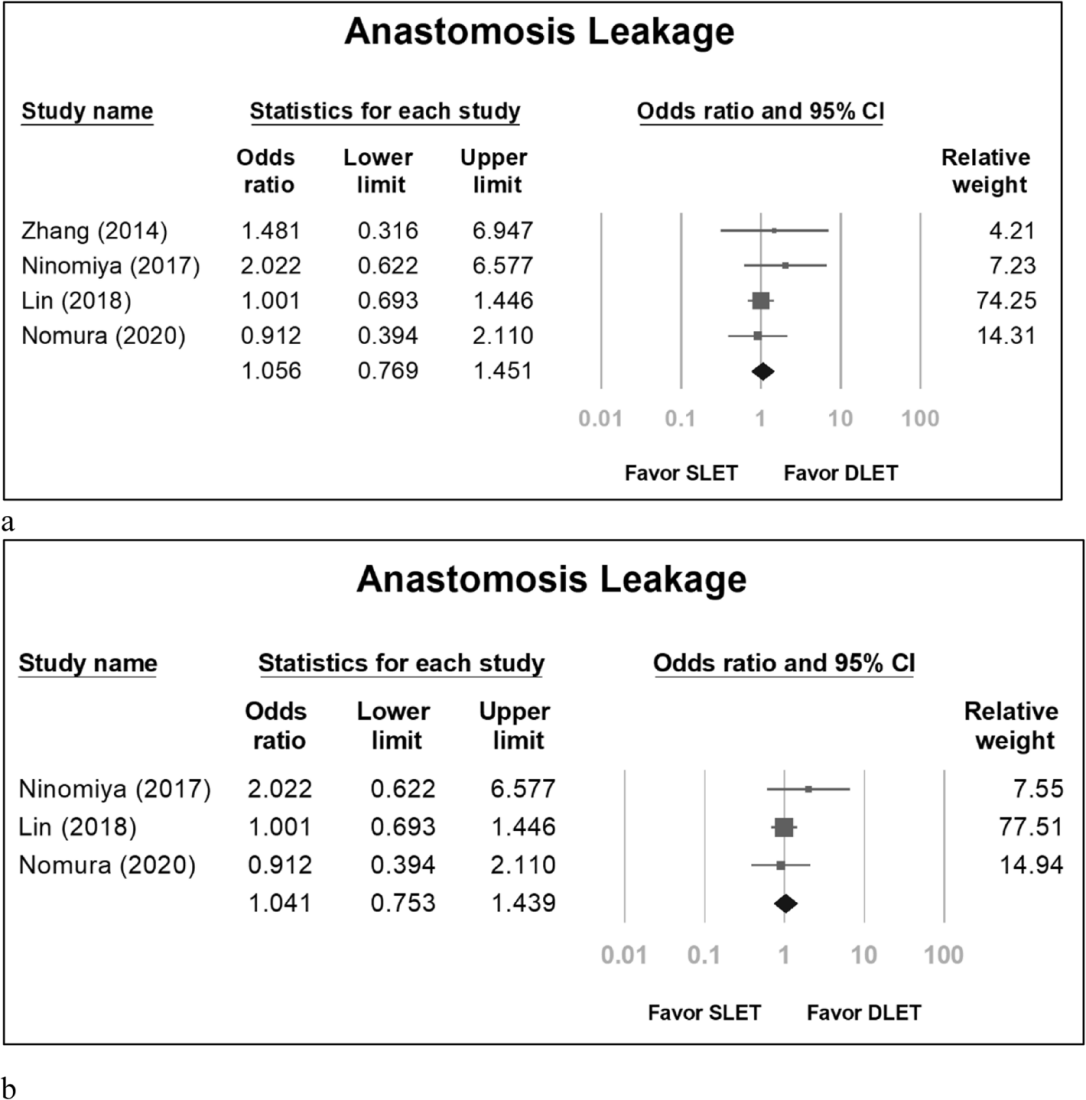
Anastomosis leakage between the DLET group and SLET group, including all studies (**a**) and excluding Zhang’s series (**b**)

**Fig. 4:**
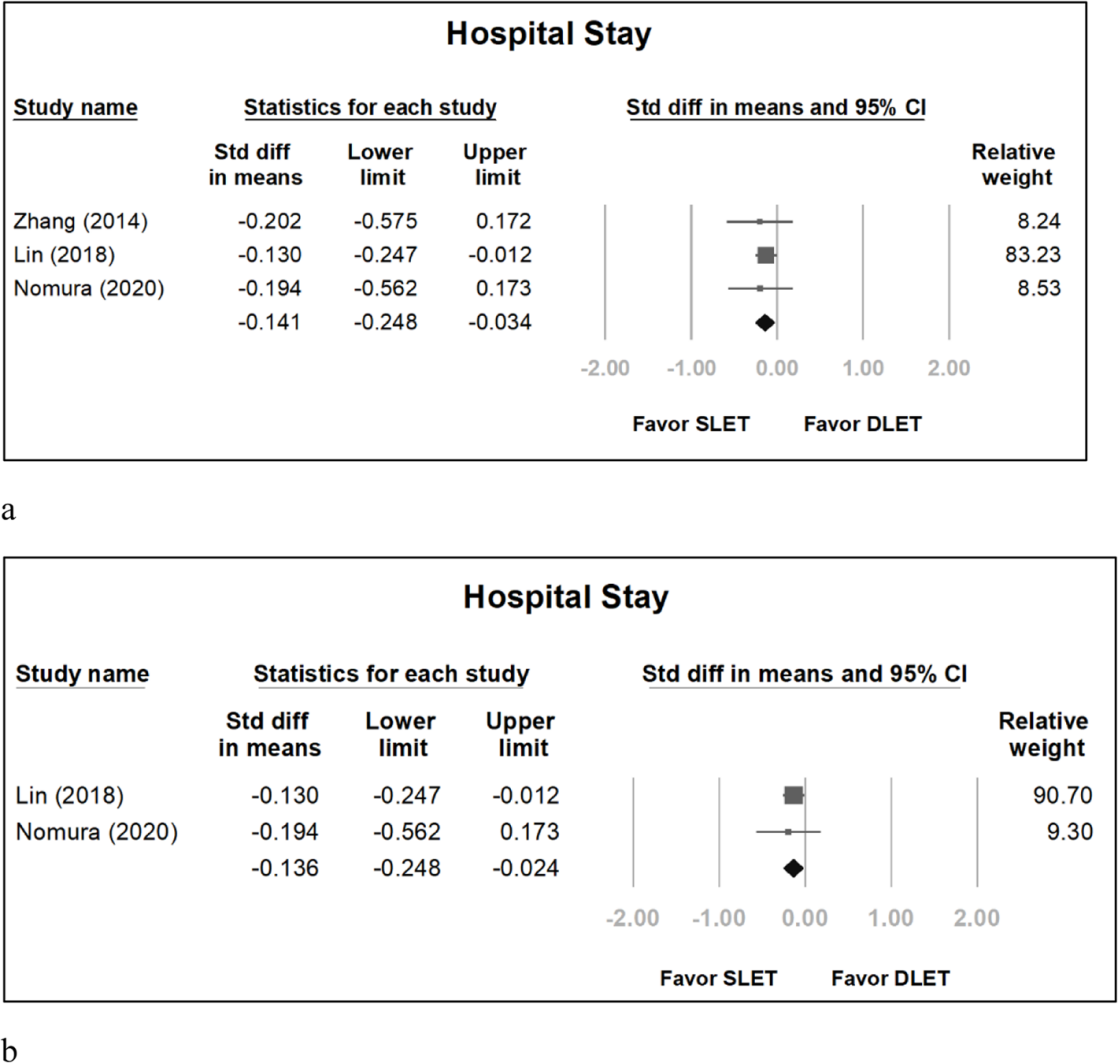
Hospitalization duration between the DLET group and SLET group, including all studies (**a**) and excluding Zhang’s series (**b**)

The pooled odds ratio for pulmonary complications in SLET group versus DLET group was 0.756 (95% confidence interval [CI]: 0.518 to 1.103) (Fig. 2a). If we excluded the study by Zhang [1] because all groups in the study used CO_2_ inflation, the pooled odds ratio was 0.775 (95% CI: 0.520 to 1.154) (Fig. 2b). The pooled odds ratio for anastomosis leakage in SLET group versus DLET group was 1.056 (95% CI: 0.769 to 1.451) (Fig. 3a). If we excluded the study by Zhang [1], the pooled odds ratio was 1.041 (95% CI: 0.753 to 1.439) (Fig. 3b). The standardized mean difference (SMD) concerning hospitalization duration in SLET group versus DLET group was − 0.141 (95% CI: − 0.248 to − 0.034) (Fig. 4a). If we excluded the study by Zhang [1], the SMD was − 0.136 (95% CI: − 0.248 to − 0.024) (Fig. 4b).

**Fig. 5:**
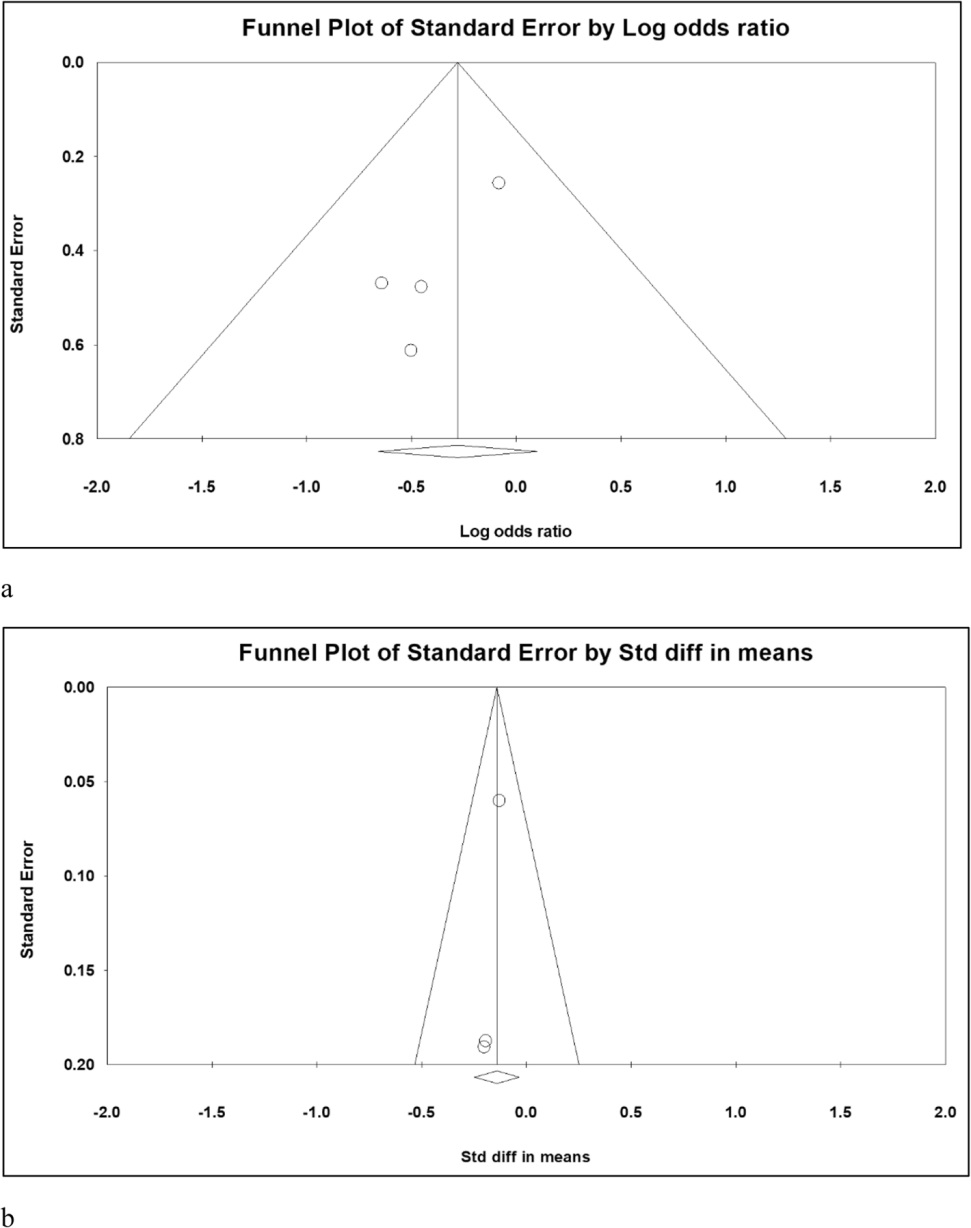
Funnel Plot of pulmonary complication studies (**a**) and hospitalization duration studies (**b**)

The Egger’s test revealed no significant publication bias concerning the overall odds ratio for pulmonary complications and anastomosis leakage (*p* = 0.348) and overall SMDs in hospitalization duration (*p* = 0.023). The funnel plots for log odds ratio for pulmonary complications and SMD in hospitalization duration are shown in Fig. 5a and b, respectively.

## Discussion

Esophagectomy is a complicated surgery which leads to high post-operative complications, morbidity, and mortality. Although minimally invasive esophagectomy can decrease hospitalization duration, it is still associated with a high post-operative risk. Double-lumen intubation with one-lung ventilation to get better surgical field exposure is a widely used anesthesia strategy, but it increases the rate of acute lung injury and increases pulmonary complications. It is difficult to perform and maintain induction and intubation with this strategy [18]. Double lumen endotracheal tube intubation and one lung ventilation may provide better surgical working field. However, this procedure need perform under bronchoscope to set exact tube position which is higher cost than single lumen endotracheal tube intubation. One lung ventilation method also easily led to barotrauma of dependent lung and lung atelectasis in the working site. These problems will increase complication after procedure [19]. Owing to the difficult manipulation regarding ventilation setting, post-operative pulmonary complications occur frequently in procedures with an extended surgical duration, such as esophagectomy [20].

Some surgeons have tried to use CO_2_ inflation to induce artificial pneumothorax and prevent one-lung ventilation. Most of these studies were case control series comparing double-lumen endotracheal tube intubation with one-lung ventilation to single-lumen endotracheal tube intubation with two-lung ventilation and CO_2_ inflation. All of them showed similar results in DLET and SLET groups but failed to mention whether there were differences in the rate and severity of complications.

The present meta-analysis focuses on the pulmonary complications, anastomosis leakage, and hospitalization duration in the two groups. We included four papers related to double-lumen endotracheal tube intubation and single-lumen endotracheal tube intubation. One of these studies [1] used CO_2_ inflation in both groups, whereas the others used CO_2_ inflation in the SLET group alone.

Compared with the DLET group, we found fewer pulmonary complications and shorter hospitalization duration in the SLET group, but the difference was not significant. In the comparison of anastomosis leak, we did not see better results in the SLET group. In these studies, the SLET group maintained ventilation in both lungs and prevented one-lung ventilation, which causes oxidative stress, capillary shear stress, and reperfusion injury. This may lead to better results in terms of pulmonary complications and hospitalization duration. As pulmonary complications improved and systemic inflammation reactions decreased [21], anastomosis leakage should also improve. However, this was not observed in the present analysis. Different surgeons bias may lead to the strange results in anastomosis leakage.

There are several limitations to our meta-analysis. First, all the included papers were case control studies. Plenty of bias could be predicted in these studies and lacked detail pre-operative parameters for analysis. No randomized control trial or prospective study can be conducted into meta-analysis for better evidence. These problems will decrease the evidence level of the meta-analysis. Second, each study used different CO_2_ inflation strategies; one even used CO_2_ inflation in both comparison groups. Meta-analysis is focused on published literatures. If these literatures did not list these preoperative parameters, we could not perform analysis. We will add limitation in the discussion to talk about this problem. We also tried to request raw data by contact with authors, but no one responded. Without raw data, we could not know these patients’ characters before surgical intervention and bias will conduct into our analysis. Third, pulmonary complications are difficult to define clearly. We found that pulmonary complications included thorax infection, pulmonary infection, and pulmonary atelectasis in the study by Zhang [1]; respiratory complications in the study by Miao [14]; pneumonia, atelectasis, and ARDS in the study by Ninomiya [15]; and respiratory complications in the study by Shinsuke [17]. Each of them defined pulmonary complications differently, which may have increased the bias of the results.

Based on these limitations, future research involving esophagectomy under SLET should focus on a randomized control study and define protocols of CO_2_ inflation and criteria for pulmonary complications.

## Conclusions

In this meta-analysis, single-lumen intubation anesthesia with artificial pneumothorax induced by CO_2_ inflation was observed to be a better option for minimally invasive esophagectomy. The odds ratio for pulmonary complications and SMDs in hospitalization duration decreased in this surgical setting. Therefore, our meta-analysis survey suggested that single-lumen intubation with CO_2_-induced artificial pneumothorax should be taken into consideration for minimally invasive esophagectomy.

## Data Availability

All data generated or analysed during this study are included in this published article (and its supplementary information files.)

## Abbreviations

VATS: Video-assisted thoracoscopic surgery
CI: Confidence interval
DLET: Double-lumen endotracheal tube intubation
SLET: Single-lumen endotracheal tube intubation
SMD: Standardized mean difference
